# Two-stage revision in periprosthetic joint infection of the hip using a new intraoperatively molded articulating spacer design

**DOI:** 10.1016/j.jcot.2023.102223

**Published:** 2023-07-16

**Authors:** Giorgio Cacciola, Fortunato Giustra, Francesco Bosco, Federico De Meo, Antongiulio Bruschetta, Pietro Cavaliere

**Affiliations:** a University of Turin, Centro Traumatologico Ortopedico (CTO), Department of Orthopaedic Surgery, Via Gianfranco Zuretti, 29, 10126, Turin, Italy; b Department of Orthopaedics and Traumatology, Ospedale San Giovanni Bosco di Torino, ASL Città di Torino, Turin, Italy; c Istituto Ortopedico del Mezzogiorno d’Italia “Franco Scalabrino”, Via Consolare Pompea, 98100, Messina, Italy

**Keywords:** Two-stage, Total hip arthroplasty, THA, Periprosthetic joint infection, PJI

## Abstract

**Purpose:**

The treatment of periprosthetic joint infection (PJI) after total hip arthroplasty (THA) is still under debate. Two-stage exchange arthroplasty is the most common surgical procedure performed. This study analyzed a new functional articulating hip spacer called "Spaceflex" regarding the mechanical-related complications, the recurrent/persistent infection during the interval period, the overall infection-free survivorship after reimplantation, the overall complication/reoperation after reimplantation and the evaluation of patient-reported outcome measures (PROMs) at three intervals of time: before the first stage, during the interval period, and at the final follow-up after reimplantation.

**Methods:**

A consecutive series of 56 patients with chronic hip PJI undergoing two-stage prosthetic revision using a new intraoperatively molded articulating hip spacer design implanted by the same experienced surgeons was examined from January 2017 to December 2021. The demographic and clinical characteristics of the included patients were analyzed. Specifically, PROMs before the first stage, during the interval period, and at the final follow-up after reimplantation and complications reported during the interval period and after reimplantation were examined.

**Results:**

The new functional articulating hip spacer was characterized by a low mechanical complication rate (5.8%) and an overall two-stage procedure success rate of 90.6% at the last follow-up. PROMs improved with the spacer during the interval period and at the final follow-up. Furthermore, the reinfection rate was in line with other case series with different spacer designs. Finally, low postoperative complication rates after reimplantation have been demonstrated.

**Conclusions:**

Two-stage revision performed with a modular articulating spacer allows patients to preserve satisfactory functional and quality-of-life outcomes in the postoperative period, with a low risk of mechanical complications and without increasing the reinfection rate.

## 1 Introduction

Periprosthetic joint infection (PJI) is a rare and devastating complication that occurs in 0.7%–2.5% of patients undergoing total hip arthroplasty (THA)^1^^,^^2^. Despite continuous improvements in surgical techniques, implants, early diagnosis, and treatment, hip PJI has not decreased in recent years^2^^,^^3^. The PJI treatment is still under debate because these complications are characterized by high management costs and the risk of poor outcomes^4^^,^^5^. Two-stage exchange arthroplasty is the most common surgical procedure for treating chronic infections, and it is often considered the "gold-standard" technique for almost all PJI^6^^,^^7^. It is the treatment of choice also in cases of sepsis manifestations, negative preoperative cultures, multiresistant organisms, and sinus tract presence^6^^,^^7^. Other possible surgical options include irrigation, debridement with or without polyethylene exchange, or one-stage exchange arthroplasty, which are viable options in selected patients^6^^,^^7^.

Historically, static spacers were used only to provide an adequate local antibiotic concentration to eradicate infection^8^^,^^9^. Several recent studies demonstrated that articulating hip spacers were characterized by better function, improved range of motion (ROM), and greater patient satisfaction than simple static block spacers^8^^,^^9^. In recent decades, articulated spacers have become increasingly popular, undergoing considerable technological developments. Therefore, the indication for static spacers should be limited to patients with significant bony defects or lack of abductor mechanism^9^. Currently, at least three different types of articulating spacers can be used during revision hip surgery: handmade, preformed/prefabricated, and functional articulating^10^^,^^11^. However, although many studies tried comparing the outcomes and complication rates of different devices, there is still no consensus on which articular spacer is the most appropriate^10^^,^^11^. On the other hand, mechanical complications, such as dislocation or spacer and periprosthetic femur fractures, occur more frequently with articulating spacers, especially in handmade ones^12^.

A new functional articulating hip spacer, presented in this study and called "Spaceflex", consists of a central titanium structure covered with antibiotic-loaded bone cement. The rationale for this spacer development is to reduce the risks of mechanical complications associated with articulating spacers, allow partial weight-bearing throughout the interval period, and customize antibiotic therapy by mixing antibiotic powder during spacer preparation.

The primary aims were to investigate: (1) the mechanical-related complications, (2) the recurrent/persistent infection during the interval period, and (3) the overall infection-free survivorship after reimplantation. The secondary aims were to analyze (4) the overall complication/reoperation after reimplantation and (5) the evaluation of patient-reported outcome measures (PROMs) at three intervals of time: before the first stage, during the interval period, and at the final follow-up after reimplantation.

## 2 Methods

### 2.1 Search strategy

A retrospective study was performed on a consecutive series of 56 patients with chronic hip PJI undergoing two-stage prosthetic revision using a new intraoperatively molded articulating hip spacer design (Spaceflex hip, G21, San Possidonio, Mo, Italy). Spaceflex hip G21 spacers were implanted by the same experienced surgeons at the Franco Scalabrino Orthopaedic Institute of Southern Italy from January 2017 to December 2021.

### 2.2 Inclusion and exclusion criteria

**Fig. 1:**
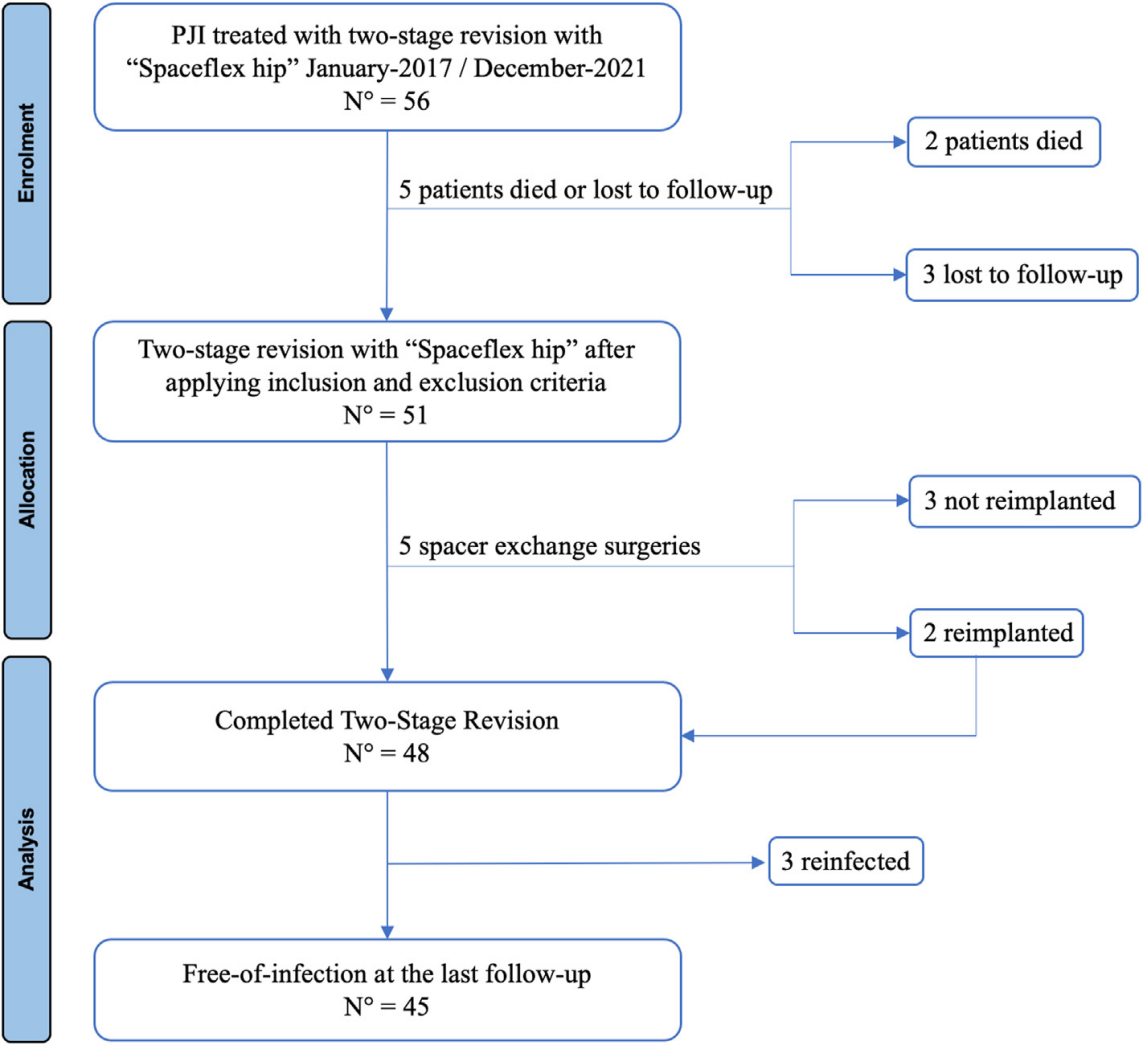
Consort Flow diagram of the study. PJI: Periprosthetic joint infection; N°: Number of evaluations cases.

All included patients met the criteria for diagnosis of chronic hip PJI, according to the PJI criteria of the International Consensus meeting^13^^,^^14^. No other types of preformed or handmade spacers were in the included patients, regardless of the extent of the bony defect at either the acetabular or femoral level^13^^,^^14^. Patients with no radiographs before the resection arthroplasty, during the interval period, or after the reimplantation were excluded from the study. During the study period, two patients died from causes unrelated to the surgery within one year after surgery, while three patients were lost during follow-up and were excluded from the study. Fifty-one patients were included in the final analysis with a mean follow-up of 2.8 (1-5) years. Forty-eight of them completed the two-stage revision, while five retained spacers. A Consort Flow diagram of the study was reported in Fig. 1.

### 2.3 Data extraction

The demographic and clinical characteristics of the included patients were analyzed. The mean number of previous surgeries and microbiologic characteristics resulting from culture examinations performed on preoperative synovial aspiration were evaluated. Furthermore, PROMs before the first stage, during the interval period, and at the final follow-up after reimplantation and complications reported during the interval period and after reimplantation were reported.

### 2.4 Surgical technique

#### 2.4.1 First stage

**Fig. 2:**
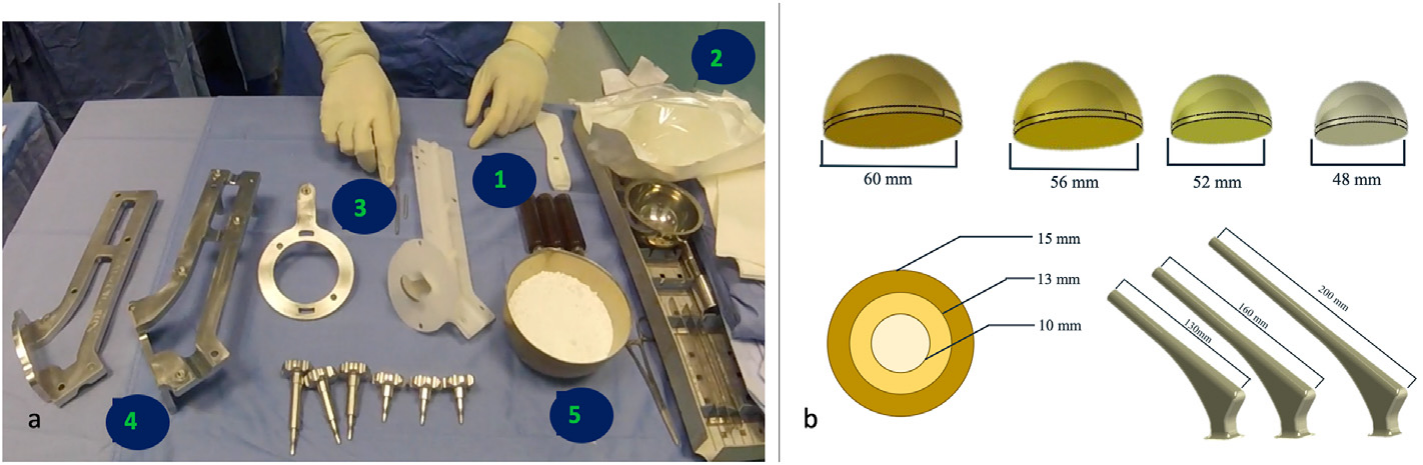
Fig. 2a: Spacer preparation instrumentation. (1) mold for the femoral part of the spacer, (2) mold for the acetabular part of the spacer, (3) short and long titanium core extension to increase the length of the femoral stem, (4) press, (5) 120 g of G3a bone cement. Fig. 2b: Modularity of the spacer. During spacer preparation, the surgeon can choose from four femoral head diameters (48 mm–60 mm), three femoral stem diameters (10 mm–15 mm) and three different stem lengths (130 mm–200 mm).

A standard postero-lateral approach to the affected hip was performed in all patients. After extensive debridement and removal of all infected materials, five soft tissue samples were collected for microbiological and histological analysis. The custom-made articulating spacer was then created with antibiotic-preloaded Polymethyl methacrylate (PMMA), named G3A cement (G21, San Possidonio, Modena, Italy), and consisting of 40g low viscosity cement with the addition of 2.5% gentamicin^15^. PMMA was supplemented with a second antibiotic specifically for the antibiogram when possible. Depending on the patient's anatomy, different sizes and lengths of the femoral head and stem are possible. Fig. 2 shows the preparation technique of the Spaceflex hip G21 spacer. In the case of a significant bone defect at the femoral metaphysis level, the spacer's neck was cemented to provide greater rotational stability and avoid loosening^16^. Cement is introduced during the kneading phase to prevent it from penetrating the bone, thus ensuring strong fixation^17^.

#### 2.4.2 Interval period

**Fig. 3:**
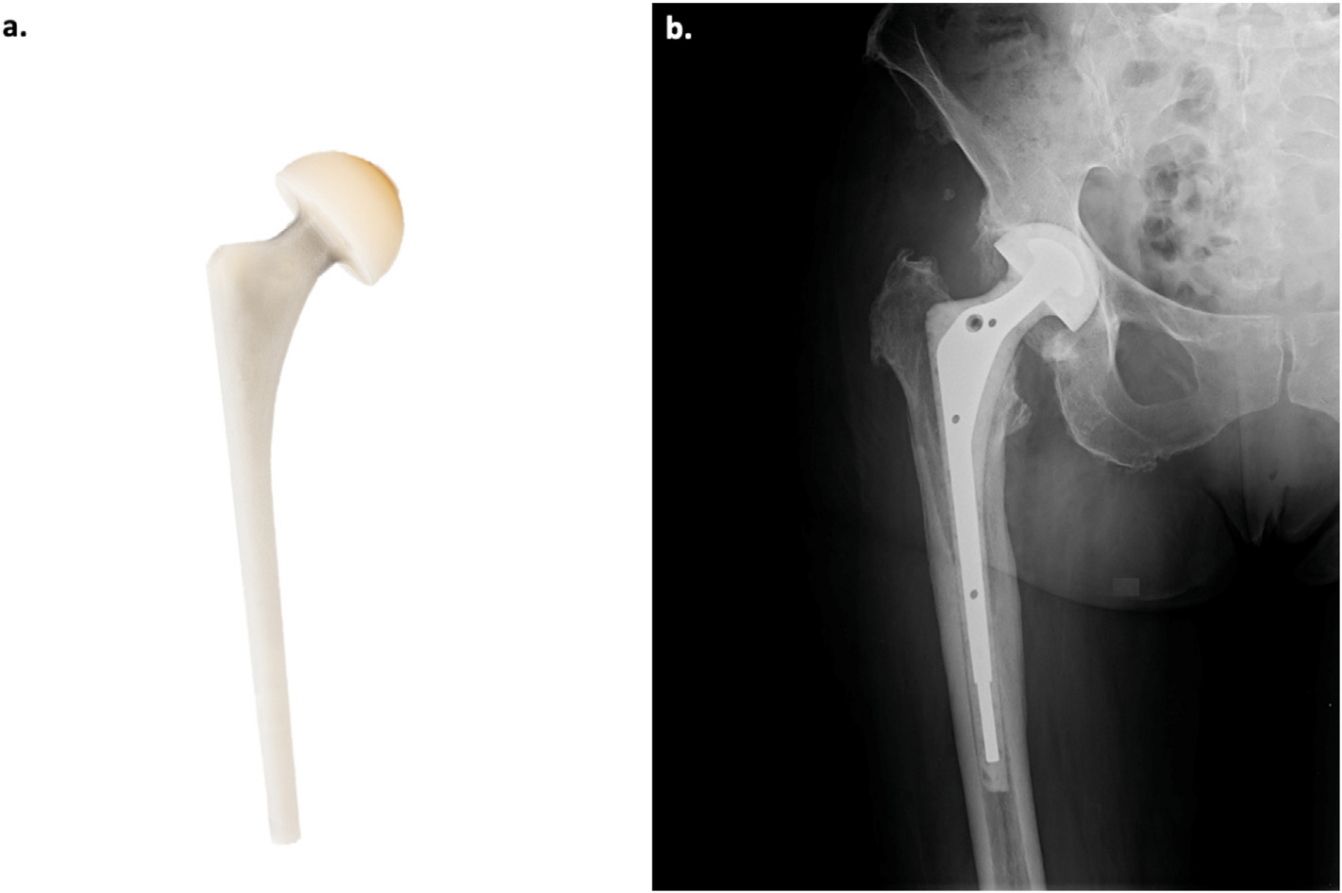
Fig. 3a: 3D model of the Spaceflex hip G21 spacer. Fig. 3b: An anteroposterior (AP) X-ray of the right hip showing an implanted Spaceflex in the immediate postoperative during the interval period of a two-stage revision.

Partly weight-bearing was recommended for all patients to reduce spacer-related mechanical problems during the interval period. In the case of a recognized pathogen, specific antibiotic therapy was started on the same day as the first stage based on the preoperative antibiogram. In case of a negative culture result, empiric broad-spectrum antibiotic therapy with gentamicin and vancomycin was started^18^. If gentamicin resistance was detected, an alternative antibiotic from the same class of aminoglycosides, but with higher sensitivity according to the antibiogram, was utilized. In case of isolation of a different or new microorganism from the tissue collected during the first stage, therapy was changed according to the information from the new antibiogram. At least two weeks of intravenous therapy was usually performed, followed by a minimum of four weeks of oral therapy. Antibiotic therapy was discontinued for two weeks if clinical and serum signs were negative for infection^19^^,^^20^. If clinical and serum negativity for infection was confirmed after about 3–4 months, reimplantation was performed. If clinical and serum signs of infection persisted 3–4 months following spacer implantation surgery, a new spacer replacement surgery was performed with the same surgical technique as described. Fig. 3 shows a 3D model of the Spaceflex hip G21 spacer and the radiographic aspect in an X-ray obtained in the immediate postoperative during the interval period of a two-stage revision.

#### 2.4.3 Second stage

**Fig. 4:**
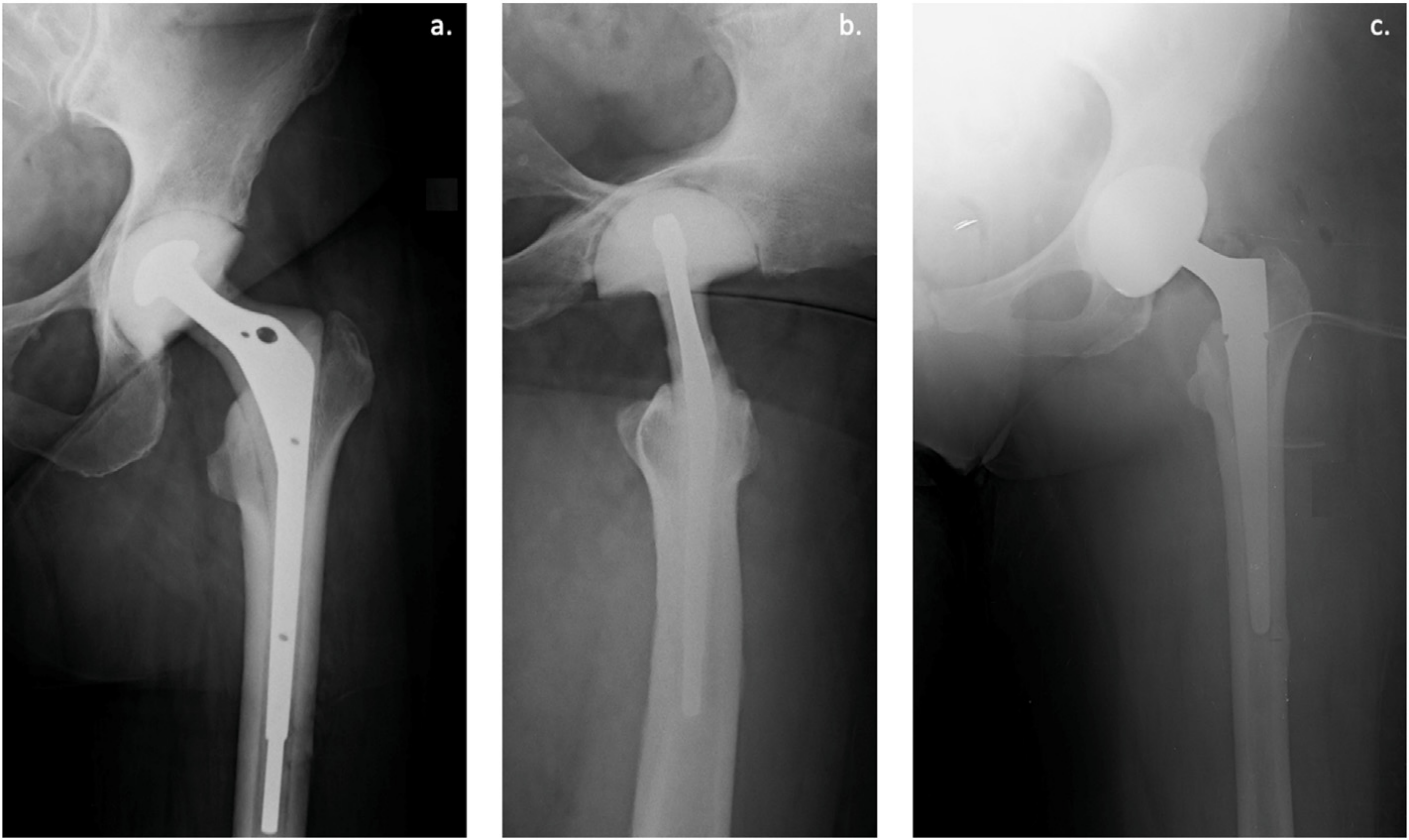
Fig. 4a: An anteroposterior (AP) X-ray of the left hip shows an implanted Spaceflex in the immediate postoperative during the interval period. Fig. 4b: An axial X-ray of the left hip shows an implanted Spaceflex in the immediate postoperative during the interval period. Fig. 4c: An AP X-ray of the left hip shows an implanted Spaceflex in the immediate postoperative reimplantation at the second stage.

All surgeries were performed with the same postero-lateral approach as the first stage. The spacer was removed, and extensive irrigation and debridement of soft tissue and dead bone were performed. Before reimplantation was performed, five tissue samples were collected for microbiological analysis. The femoral and acetabular components used for reimplantation differed depending on the extent of the bone defect or muscle deficiency. In all cases, acetabular and femoral press-fit components were used. A constrained acetabular component was implanted only in complete adductor muscle deficiency cases. Postoperative antibiotic therapy was not routinely performed. Targeted therapy was performed in case of persistent positive culture results after reimplantation^21^. Postoperative X-rays were obtained at the end of the spacer implantation during the interval period and after the reimplantation at the second stage to confirm the correct positioning of the implanted components (Fig. 4).

### 2.5 Ethical approval

The author's institution's Institutional Review Board (IRB) defined this study as exempt from IRB approval (retrospective study of an established surgical procedure). It was conducted following the ethical standards of the Declaration of Helsinki (1964).

### 2.6 Statistical analysis

Descriptive statistical analysis was performed for all data extracted from the included studies using IBM SPSS Statistics (version 25.0). For continuous variables, mean values were calculated or extracted with a measure of variability as standard deviation (SD) or range (minimum-maximum). For categorical variables, the absolute number and frequency distribution were calculated. Patient-reported outcome questionnaires were compared with the Friedman test before explantation, with spacer, and after reimplantation. Final implant revision rates were calculated using Kaplan-Meier survival analysis. A p-value <0.05 was considered statistically significant.

## 3 Results

### 3.1 Demographics

The baseline diagnoses of chronic hip PJI that led to the two-stage revision were primary THA infection in 41 patients and revision THA infection in 10 cases. The demographic and clinical characteristics of the 51 patients included in the final analysis are listed in Table 1. The result of microbiological culture growth obtained through preoperative synovial aspiration is shown in Table 2.

**Table 1:** Baseline characteristics of the population undergoing two-stage revision using Spaceflex hip G21 spacer.

**Demographic data**	
Total N° of hips; N° (%)	56 (100%)
Died or lost to follow-up; N° (%)	5 (8.9%)
Average age (years); mean ± SD	72.2 ± 11.4
Gender, female; N° (%)	35 (68.6%)
Average BMI (Kg/m^2^); mean ± SD	28.4 ± 4.9
**Clinical characteristics**	
Average N° of previous surgeries; mean ± SD	2.2 ± 1.3
Average Carlson Comorbidity Index; mean ± SD	3.7 ± 1.5
Average ASA score; mean ± SD	2.9 ± 1.3
Infection of primary THA; N° (%)	41 (80.3%)
Infection of revision THA; N° (%)	10 (19.7%)
**Preoperative clinical scores**	
Average Preoperative HHS; mean ± SD	38.7 ± 17.9
Average Physical SF-12; mean ± SD	24.2 ± 8.8
Average Mental SF-12; mean ± SD	22.3 ± 8.1

N°: Number of evaluation cases; %: percentage; SD: standard deviation; Kg/m^2^: Kilogram/meter^2^; ASA: American Society of Anesthesiologists; THA: Total hip arthroplasty; HHS: Harris Hip Score; SF-12: The 12-item Short Form Survey.

**Table 2:** Microorganisms identified by preoperative synovial aspiration.

Causative microorganism	N° (%)
S. Epidermidiis	27 (48.2%)
S. Aureus	12 (21.4%)
MRSA/MRSE	4 (7.1%)
K. Pneumonia	2 (3.6%)
E. Coli	2 (3.6%)
Polymicrobial	5 (8.9%)
Culture negative	4 (7.1%)

N°: Number of evaluations cases; %: percentage.

### 3.2 Complications during the interval period

The overall rate of mechanical complications was 5.8% (3 cases out of 51). Two cases of spacer dislocation (3.9%) were reported, one managed with closed reduction and one treated with spacer replacement surgery due to recurrent dislocation. The femoral head diameter was changed from 48 mm to 52 mm; no dislocation occurred after the spacer replacement. Another patient showed a peri-spacer fracture of the greater trochanter, which was treated by limiting weight bearing and avoiding hip flexion more than 90° for four weeks. No spacer fractures were reported. Five patients showed signs of persistent infection during the interval period (9.8%). Two of them were free of infection six weeks after the spacer replacement surgery and were reimplanted. Two showed recurrent signs of infection after spacer replacement surgery and were managed with chronic antibiotic therapy. One of them showed no signs of infection but refused reimplantation surgery. For these patients, spacer replacement surgery was performed with a technique the same as previously described. The overall survival without infection during the interval period, considering patients who recovered after spacer replacement surgery, was 96.1%. The overall complication rate during the interval period was 15.6%, while the reimplantation rate was 94.2% after a mean interval period of 9.8 (8 -17) weeks.

### 3.3 Complications after reimplantation

Overall survivorship of 90.6% (43 of 48 cases) was found after a mean follow-up of 2.8 (1-5) years. One case of early loosening of the femoral stem after 4.5 months following reimplantation was evidenced, which underwent single-stage revision. One patient presented with recurrent dislocation in which the acetabular component was revised to achieve greater anteversion in the immediate postoperative period. Three patients showed postoperative signs of recurrent infection 3 months, 4.5 months, and 16 months after reimplantation, respectively. In all these cases, a two-stage revision was performed again. Three other minor complications were reported that did not require component revision. One episode of dislocation managed with a closed reduction in two patients and one case of wound dehiscence in one patient managed with oral antibiotic therapy were reported. A summary of complications reported during the interval period and after reimplantation was given in Table 3.

**Table 3:** Complications were reported during the interval period, and complications were reported after reimplantation.

Complications	During interval period (Total N° hips: 51)		After reimplantation (Total N° hips: 48)	
	Overall, N° (%)	Spacer exchange surgery, N° (%)	Overall, N° (%)	Revision surgery, N° (%)
Dislocations	2 (3.9%)	1 (1.97%)	2 (4.2%)	1 (2.1%)
Periprosthetic fracture	2 (3.9%)	0 (0%)	0 (0%)	0 (0%)
Recurrent infection	5 (9.8%)	5 (9.8%)	3 (6.3%)	3 (6.3%)
Wound dehiscence	0 (0%)	0 (0%)	1 (2.1%)	0 (0%)
Aseptic loosening	0 (0%)	0 (0%)	1 (2.1%)	1 (2.1%)
Overall	9 (17.6%)	6 (11.7%)	7 (14.6%)	4 (10.4%)

N°: Number of evaluation cases; %: percentage.

### 3.4 PROMs

PROMs measured before surgery, during the interval period, and at the last follow-up were reported in Table 4. Mean Harris Hip Score (HHS) improved significantly from a mean of 38.7 ± 17.9 before the first stage to a mean of 63.3 ± 19.4 during the interval period (p < 0.034) and to 78.3 ± 17.9 at the last follow-up after reimplantation (p < 0.003). The Forgotten Joint Score (FJS) was recorded after reimplantation with a mean score of 74.2 ± 21.1. Regarding the physical subscale of the 12-item Short Form Survey (SF-12), there was a significant improvement from a mean preoperative score of 24.2 ± 8.8 to a mean score of 33.5 ± 9.1 during the interval period (p < 0.031) and to a mean score of 44.4 ± 8.1 at the last follow-up (p < 0.002). Similarly, there was a significant improvement in the mean mental subscale of the SF-12 from 22.3 ± 8.1 before the intervention to a mean score of 29.9 ± 9.3 during the interval period (p < 0.004) and to a mean score of 41.3 ± 11.1 at the last follow-up (p < 0.029).

**Table 4:** PROMs were measured before surgery, during the interval period, and at the last follow-up. Statistically significant differences were reported in bold.

PROMs	Preoperative values	Interval Period values	Last follow-up values	P-value preoperative vs interval period/interval period vs last follow-up
	Mean ± SD	Mean ± SD	Mean ± SD	P-value/P-value
HHS	38.7 ± 22.4	63.3 ± 19.4	78.3 ± 17.9	0.064/**0.003**
FJS	N/A	N/A	74.2 ± 21.1	N/A
Physical SF-12	24.2 ± 8.8	33.5 ± 9.1	44.4 ± 8.1	0.051/**0.002**
Mental SF-12	22.3 ± 8.1	29.9 ± 9.3	41.3 ± 11.1	0.064/**0.029**

HHS = Harris Hip Score; FJS = Forgotten Joint Score; SD: standard deviation; N/A: Not available; SF-12: The 12-item Short Form Survey.

## 4 Discussion

Two-stage revision remains the "gold standard" treatment in chronically infected THA^5^^,^^6^. Good results are reported in the literature using different static and articulating spacers; however, one of the main concerns is the high mechanical complications rate, described from 0% to 70%^22^, ^23^, ^24^. The Spaceflex hip G21 spacer was characterized by low mechanical complications rate of 5.8% and an overall two-stage procedure success rate of 90.6% at the last follow-up. Finally, all the PROMs improved with the spacer during the interval period and at the final follow-up.

### 4.1 Complications during the interval period

The mechanical complications incidence during the interval period is not well defined in the literature and varies from 0% to 70% according to the different case series^22^, ^23^, ^24^. Erivan et al., in their study of 26 patients, reported a mechanical complication rate of 73%, where spacer dislocation was the most frequent complication^22^. Faschingbaur et al. described at least one mechanical complication in 19.6% of patients who underwent a two-stage procedure, with equal 8.9% of both dislocation and spacer fracture^23^. Jung et al. reported a mechanical complication in 45.1% of cases; dislocation and spacer fracture were the most frequent, with an incidence of 18.3% and 10.9%, respectively^24^.

Recent studies suggest avoiding prolonging the interval period to reduce mechanical complications and infection recurrence risks^24^^,^^25^. In a recent systematic review that analyzed mechanical complications observed during the interval period, the authors reported a weighted mean of 19% for mechanical complications^12^. Spacer dislocation was the most frequent one described in 10.8% of cases. Peri-spacer and spacer fractures accounted for 3.5% of cases, respectively. Finally, "acetabular complications," such as acetabular osteolysis and pelvic protrusion, were described in 1.2% of cases^12^. In this study, the complication rate reported using the Spaceflex hip G21 spacer is significantly lower than that reported in the literature. Spacer dislocation was this series' most frequent mechanical complication, with an incidence of 3.9%. In the two cases described, one patient was treated by closed reduction, while the other required reoperation with spacer replacement. This last patient was a 47-year-old man with a BMI of 41, significant adductor muscle weakness, four previously failed surgeries, two DAIRs and a two-stage revision. In this case, the femoral head size was increased from 48 mm to 52 mm, and the femoral neck was cemented to avoid rotational instability. The opportunity to choose from four different femoral head sizes, three stem lengths, and diameters allows the surgeon to select a spacer that may optimally conform to each patient's specific anatomy. The only other mechanical complication reported in this series was an asymptomatic peri-spacer compound fracture of the greater trochanter, treated exclusively by restricting weight bearing and flexion over 90° for four weeks. No spacer fracture was noted in this study, although weight-bearing was allowed for all patients. In addition, no periacetabular osteolysis or pelvic protrusion was described.

Several preoperative factors associated with an increased dislocation risk are described in the literature^26^, ^27^, ^28^, ^29^. Biomechanical factors such as reduced offset or leg shortening may raise dislocation risk due to inappropriate soft tissue tension^28^^,^^29^. Similarly, an undersized spacer at the acetabular or femoral side could increase the dislocation risk due to spacer instability or mobilization^12^^,^^26^^,^^27^. Adductor muscle deficit or a severe bone defect may also result in a higher dislocation rate because they are associated with greater difficulty in achieving spacer stability intraoperatively and during the interval period^28^^,^^29^.

During the interval period, five patients (9.8%) presented signs of persistent infection; all of them underwent spacer replacement surgery after an average period of 6.9 weeks from the first stage. In three of them, no signs of persistent infection were observed; two patients underwent reimplantation, while the third refused the operation, deciding to maintain the spacer. The other two patients with persistent infection were treated with long-term suppressive antibiotic therapy.

The overall survival without infection during the interval period was 96.1%, considering the patients who recovered after the spacer replacement surgery. The mean time to reimplantation after the first stage was 9.8 weeks, involving 94.2% of treated patients. Reinfection or persistent infection during the interval period is an important complication. As reported in several case series, approximately 10%–20% of patients underwent spacer replacement surgery primarily because of infection^30^, ^31^, ^32^. Gomez et al., in a two-stage series of 504 cases, performed a spacer substitution surgery in 11.9% of patients^32^. These data align with this study's 9.8% spacer replacement rate.

### 4.2 Reinfection after reimplantation

In the literature, the reinfection rate in reimplantation after hip PJI is between 5% and 25% at an average follow-up of four years^33^^,^^34^. Lange et al., in their systematic review, reported an infection-free survival in 89.4% of patients^33^. In a recent meta-analysis, Goud et al. described a 91.6% infection-free survival in more than 5000 hip PJI treated with a two-stage revision surgery^34^. The results described in the literature are consistent with those reported in this study; after reimplantation, there was a postoperative recurrent infection in only three cases with an infection-free survival rate of 94.2% at a mean follow-up of 2.8 years. In two of the three cases, reinfection was caused by the same microorganism: in the first case, the infection was caused by Methicillin-Resistant Staphylococcus Aureus (MRSA), and in the second case, by Staphylococcus Epidermidis. In the last patient, the reinfection was culture-negative at the preoperative aspiration but was positive for Staphylococcus Aureus after reimplantation.

The low reinfection rate was also achieved by a scrupulous adherence to the hospital treatment protocol based on the best available evidence in the literature^35^, ^36^, ^37^. In clinical practice, preoperative aspiration to identify the causative microorganism is mandatory. Preoperative identification of the infective microorganism is crucial in the diagnosis and treatment algorithm^36^. The opportunity to adopt targeted antibiotic therapy instead of a wide-spectrum therapy should increase the success rate of all surgical procedures^38^^,^^39^.

### 4.3 Limitations and strengths

This study presents some limitations. First, it is retrospective in design, and the nature of this study has intrinsic limits. Second, five patients were lost to follow-up; two died within one year from causes unrelated to PJI, and three were lost before one year of follow-up. Third, not all patients underwent two-stage revision as the primary procedure for PJI treatment; moreover, there was a different bone loss, and several implants were used at the revision time. This study also has several strengths. The number of patients is large enough to be compared with other similar case series. Although this is a retrospective study, all clinical data were collected prospectively, in the internal arthroplasty registry, both at the time of surgery and at each follow-up. Finally, all patients were operated on by the same surgeon, with the same surgical technique, antibiotic therapy protocol, and postoperative care.

## 5 Conclusion

The two-stage revision performed with a modular articulating spacer allows patients to preserve acceptable functional and quality-of-life scores during the interval period maintaining low mechanical complications rate without increasing the reinfection rate compared with other case series with different spacer designs. Finally, optimal PROMs and postoperative complication rates after reimplantation were reported.

## Funding

There is no funding source.

## Authors’ contributions

GC, FG, and FB have contributed substantially to conception and design, data acquisition, analysis, and interpretation. They have been involved in drafting the manuscript and revising it critically for important intellectual content, given final approval of the version to be published. They agree to be accountable for all aspects of the work in ensuring that questions related to the accuracy or integrity of any part of the work are appropriately investigated and resolved. FDM, and AB have contributed substantially to the data analysis, interpretation, and manuscript drafting. PC has made substantial contributions to concept and design the manuscript and revising it critically for important intellectual content.

## Consent for publication

All patients gave consent to publication.

## Informed consent

Informed consent was obtained from all individual participants included in the study.

## Availability of data and materials (data transparency)

Dataset analyzed in this study is available from the corresponding author on reasonable request.

## Code availability (software application or custom code)

Not applicable.

## Declaration of competing interest

The authors declare that they have no known competing financial interests or personal relationships that could have appeared to influence the work reported in this paper.
